# Supplementary material for: Generating high-order optical and spin harmonics from ferromagnetic monolayers
Source: Nat Commun. 2018 Aug 2;9:3031. doi: 10.1038/s41467-018-05535-4 (PMC6072758; doi:10.1038/s41467-018-05535-4)
Supplement: Supplementary file 1 — Supplementary Information [file 41467_2018_5535_MOESM1_ESM.pdf]

Supplementary Information on "Generating high-order optical and spin harmonics from ferromagnetic monolayers"

G. P. Zhang*

Department of Physics, Indiana State University, Terre Haute, Indiana 47809, USA

M. S. Si

Key Lab for Magnetism and Magnetic materials of the Ministry of Education,
Lanzhou University, Lanzhou 730000, China

M. Murakami

Department of Physics, Indiana State University, Terre Haute, Indiana 47809, USA

Y. H. Bai

Office of Information Technology, Indiana State
University, Terre Haute, Indiana 47809, USA

Thomas F. George

Office of the Chancellor
Departments of Chemistry & Biochemistry and Physics & Astronomy
University of Missouri-St. Louis,
St. Louis, MO 63121, USA

(Dated: June 29, 2018)

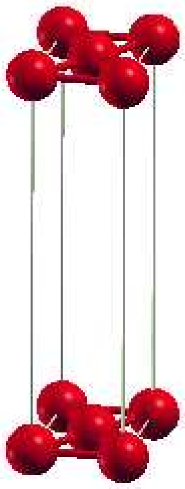

Supplementary Fig. 1: Slab geometry for Fe(110) monolayer. Our primitive cell consists of two Fe atoms: One at a corner and the other at the center.

In this supplementary information, we provide details of our calculation and extensive tests on both system-related parameters (such as k mesh size, vacuum layer thickness) and the laser-related parameters (polarizations, laser strength, photon energy and pulse duration). We also present results for both Fe (110) and (001) ultrathin films (trilayers) as well as a comparison with MoS_2.

I. SUPPLEMENTARY METHODS

To simulate Fe(110) and Fe(001) monolayers as well as ultrathin films (see below), we adopt a slab geometry (see Supplementary Fig. 1), where there is a vacuum spacing between each slab. This vacuum layer must be thick enough to ensure that the interaction two layers is extremely small. Supplementary Figure 1 shows an example for the Fe(110) monolayer. Fe(110) has a rectangular cell (see Supplementary Fig. 1), where the lattice constant along the b axis is $\sqrt{2}$ times the lattice constant along the a axis, $b = \sqrt{2}a$. Fe(001) has a higher symmetry, with the equal length of the base basis vectors.

The vector potential of the laser field for circularly polarized light is

$$\mathbf{A}(t) = A_0 \mathrm{e}^{-t^2/\tau^2} (\cos(\omega t)\hat{x} \pm \sin(\omega t)\hat{y}) \tag{1}$$

where t is the time, τ is the laser pulse duration, ω is the carrier frequency, $+$ and $-$

refer to the the left (σ^-) and right (σ^+) circularly polarized light within the xy plane, respectively. A_0 is the field amplitude in units of Vfs/Å. One can convert Vfs/Å to V/Å using E_0(V/Å) $= A_0$(Vfs/Å)ω, where ω is the laser frequency and E_0 is the field amplitude in V/Å. For instance, for our current laser field, a field amplitude of 0.03 Vfs/Å and photon energy of 2 eV corresponds to 0.09 V/Å. $\hat{x}$ and $\hat{y}$ are the unit vector along the x and y axes, respectively. For linearly polarized light along the z axis, we choose

$$\mathbf{A}(t) = A_0 \mathrm{e}^{-t^2/\tau^2} \cos(\omega t)\hat{z}, \tag{2}$$

where $\hat{z}$ is the unit vector. We employ a similar form if the laser field is polarized along other directions.

In contrast to insulators and semiconductors, one big challenge in metals is to sample a large number of k points. We have successfully developed a massively parallel code, so we can solve thousands of differential equations (Liouville equations) in parallel,

$$i\hbar\langle i\mathbf{k}|\frac{\partial\rho}{\partial t}|j\mathbf{k}\rangle = \langle i\mathbf{k}|[H_0 + H_I, \rho]|j\mathbf{k}\rangle, \tag{3}$$

where the band indices i and j run over both valence and conduction band states. The Pauli exclusion principle is respected rigorously through the commutator on the right hand side of Supplementary Equation 3. The right hand side is going to be zero if both states i and j are occupied or both are empty. This property ensures that whether one treats many-electron or single-electron systems, there is no violation of Pauli exclusion principle. In TDSE and TDDFT, a single particle wavefunction is propagated in the time domain independently, so two initially different wavefunctions may end up in the same state. In atoms and molecules, the energy levels are typically well separated energetically, so the risk to violate the Pauli exclusion principle is low. But this is no longer the case in solids. One single k point can have many bands in a narrow energy window. This is the major difference between our method and TDDFT/TDSE. The disadvantage of our method is that we have to use density matrices, instead density (diagonal of the density matrix). So there is a heavy computational load for our method. This is the price that we have to pay, in order to get physics right.

Before we move on, we would like to explain how FFT is carried out with and without window functions. We use two window functions, hyper Gaussian $\mathcal{W}_1$ and hyperbolic tangent $\mathcal{W}_2$,

$$\mathcal{W}_1(t) = \exp\left[-(at)^8 \times b\right] \tag{4}$$

where t is in the unit of fs. Two constants are chosen so we have a window across the entire data set. We find $a = 0.035/\text{fs}$ and $b = 5 \times 10^{-9}$ (no unit) to be very good for cutting off the tail of the momentum expectation value $P(t)$ while setting the leading edge at -400 fs and the trailing edge at 400 fs. Here,

$$\mathcal{W}_2(t) = (\tanh[(t + t_1) * b_1] - \tanh[(t + t_2) * b_2])/2, \tag{5}$$

where t_1 and t_2 set the centers of the cutoffs in the beginning and in the end, respectively and b_1 and b_2 are the respective widths of the leading and trailing edges. We find that $\mathcal{W}_2$ is more flexible since it has two separate control parameters. We fix $t_1 = 400$ fs, $t_2 = -400$ fs, and $b_1 = b_2 = 0.1$ fs. In many ways, these two functions are similar, but there are some differences, which we will explain this below.

II. SUPPLEMENTARY NOTE 1: FOURIER TRANSFORM WITHOUT AND WITH WINDOW FUNCTIONS

As the power spectrum of HHG depends on the Fourier transform, we feel that it is appropriate to provide some details about our Fourier transform. We take Fig. 2(a) of the main text as an example. Other cases are similar. We consider three different cases: (i) direct Fourier transform without any window function, (ii) Fourier transform with the hyperbolic tanh function (Supplementary Equation (5)), and (iii) Fourier transform with the hyper Gaussian (Supplementary Equation (4)).

Supplementary Figure 2 compares these three cases. Supplementary Figure 2(a) shows the original data for $P_y(t)$. The red line denotes the window, which is 1, so we do not use the window function to treat our original data. We see that if we do not use the window function, the power spectrum after the Fourier transform has harmonics only up to the 7th order, with a big background. Supplementary Figure 2(c) is treated with the hyperbolic tanh window function. It cuts off the tail of $P_y(t)$, which has a small value but is oscillatory. This oscillatory portion contributes a background to the Fourier transformed spectrum. We see that after we Fourier transform it to the power spectrum, the original strong background in Supplementary Fig. 2(b) is removed, and those hidden peaks show up. The highest harmonic order is 13, and we verify that there is no other peak after this for our current laser parameters. To test whether the window function changes the amplitude of the harmonics,

we draw several dashed horizontal lines and see that the amplitudes are not altered strongly. The 7th harmonic becomes slightly weaker, but that is within the experimental error bar. To understand the behavior of the window functions, in Supplementary Fig. 2(e) we use the hyper Gaussian as our window function. Supplementary Figure 2(f) is the power spectrum. We find that the quantitative agreement between the hyperbolic tangent and hyper Gaussian is quite good. This demonstrates the high accuracy of our power spectrum. There is one minor difference: The hyper Gaussian window turns to a narrow spectrum [compare the widths of Supplementary Fig. 2(d) and Supplementary Fig. 2(f)].

Another thing that we notice is that the window function tends to reduce the baseline signal to the same small level. We take Fig. 3(a) of the paper (σ^+ polarized light) as an example. Supplementary Figure 3(a) shows the time evolution of the momentum expectation value $P(t)$ between -50 fs and 50 fs, where P_y is larger than P_x. Supplementary Figure 3(b) shows that between 100 fs and 400 fs, P_x and P_y are comparable to each other, with a much smaller absolute value. If we do not use the window function, the difference between P_x and P_y is captured, where the baseline of P_y is larger than that of P_x [see the green dashed line and blue long-dashed line in Supplementary Fig. 3(c)]. But once we use the filter, we see that their difference is no longer clear [see the solid red line and red dotted line in Supplementary Fig. 3(c)]. This finding is important, since if we want to compare two power spectra quantitatively, caution is needed here.

III. SUPPLEMENTARY NOTE 2: CONVERGENCE TEST AND DEPENDENCE STUDY

A. Approximations

Calculations on high-harmonic generation in monolayers and thin films are more demanding than those in atoms. This becomes more challenging when we have a metallic system. Different from those earlier studies in semiconductors where a single k point is used, we use a large number of k points, several orders of magnitude larger. Yet, we have a limit how many points can be included in a single calculation in order to get results within a reasonable time. Thus, we have to cut off some states in particular those low-lying states. This approximation is system-dependent. We discuss them one by one.

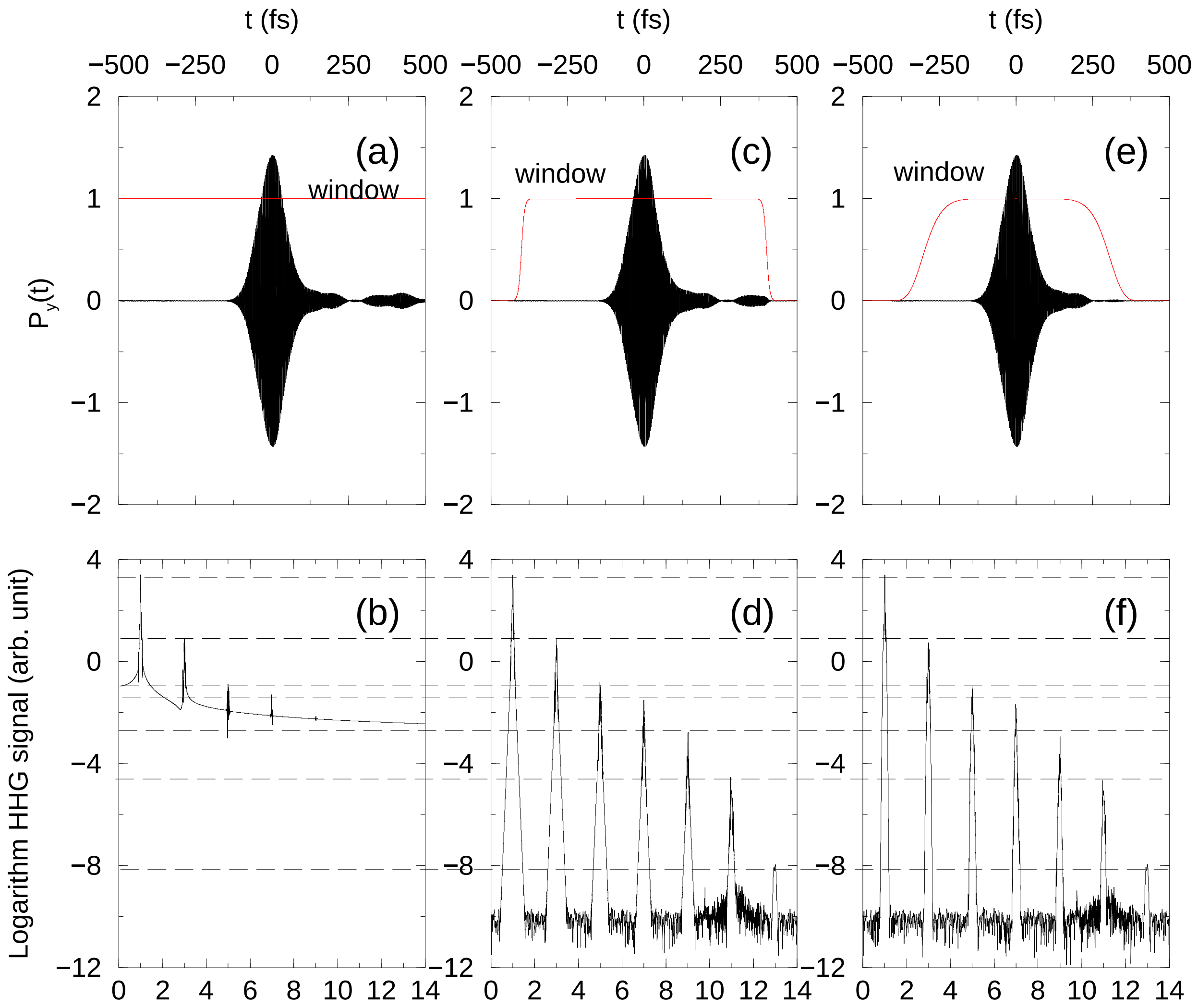

Supplementary Fig. 2: A test case for Fourier transform in the nonmagnetic Fe(110) monolayer. The original data is from Fig. 2(a) of the main text. (a) $P_y(t)$ as a function of time. The red line is the window function which is constant, or no window. (b) Power spectrum by Fourier transform from (a). The highest harmonic order is 7. (c) Same data as (a) but with the hyperbolic tanh function. Again, the red line is the window. (d) Power spectrum of (c). The highest harmonic order is up to 13. The horizontal dashed lines are drawn to show that the window function does not affect the absolute magnitude much. (e) Same as (a) but with the hyper Gaussian function. (f) Power spectrum of (e). There is good consistency in the amplitude between the hyperbolic tanh window function and the hyper Gaussian function (see the rest of the horizontal dashed lines).

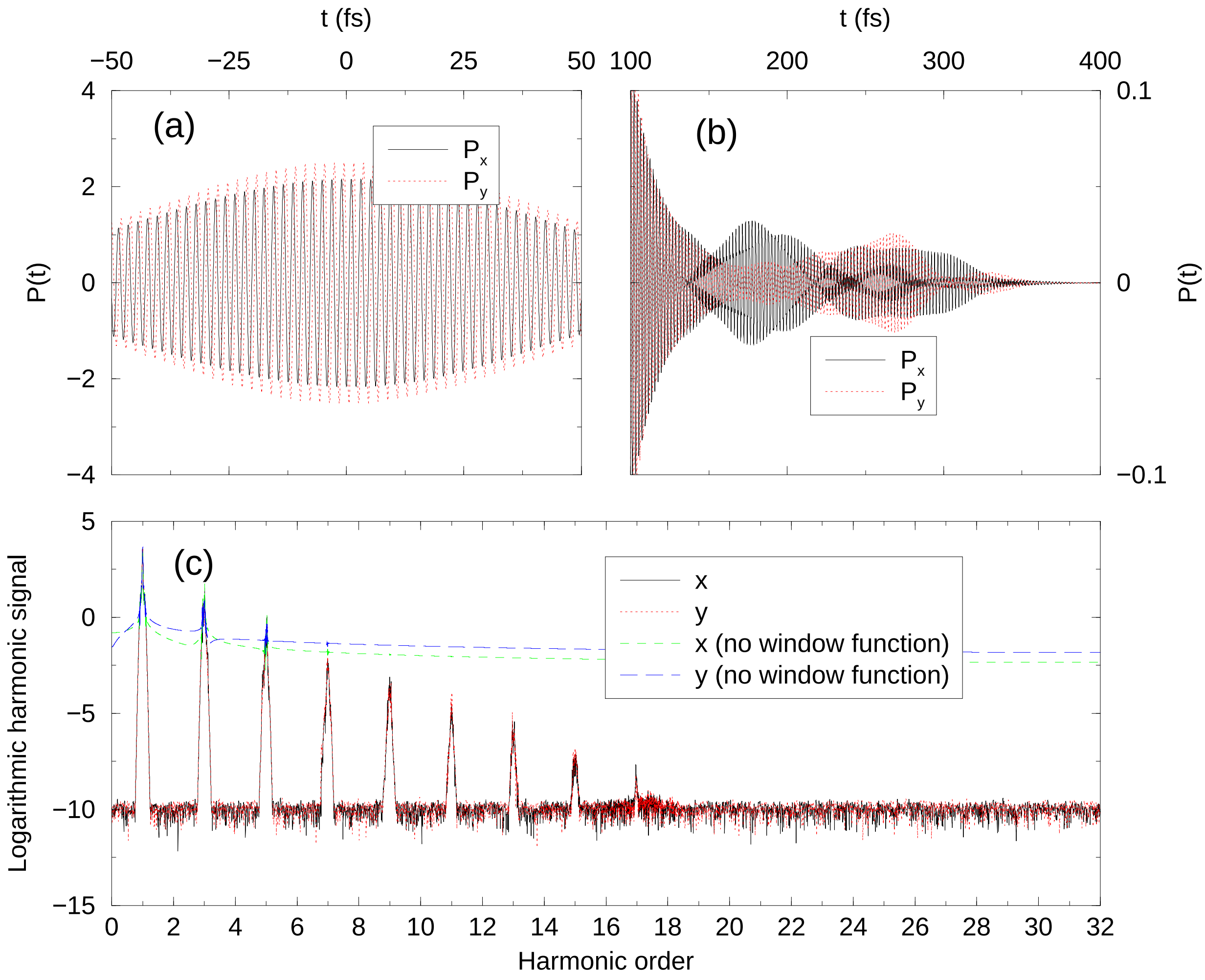

Supplementary Fig. 3: (a) Expectation value of the momentum $P(t)$ as a function of time t between -50 fs and 50 fs. The black solid curve denotes the x component, $P_x(t)$, while the red dotted curve denotes the y component, $P_y(t)$. (b) Expectation value of momentum $P(t)$ as a function of time t between 100 fs and 400 fs. (c) Logarithmic harmonic signal. The black solid line and red dotted line denote the x and y components of the spectra, computed with a window filter. The green dashed line and blue long-dashed line denote the x and y components of the spectra, computed without a window filter.

B. Fe(001) monolayer

For the Fe(001) monolayer, the Fermi energy is at -0.25 Ry. Eight very low energy states – Fe's 3s states (between -6.5 and -6.7 Ry) and 3p states (between -4.3 and -3.9 Ry) – are most time consuming and not included in our calculation. All the eight other valence

states (such as $3d$ states) are included. The entire simulation includes 108 band states (from 9 to 116) at each k point, with energy spanning from -0.58 Ry to 1.52 Ry. Our number of states is much larger than a prior calculation in GaSe where only five bands were included in Supplementary reference [1]). This is also true for another calculation carried out so far in Supplementary reference [2]. We use the same set of laser parameters as the main paper, $E_0 = 0.09$ V/Å, $\tau = 60$ fs, and $\hbar\omega = 2.0$ eV. Supplementary Figure 4(a) shows the spectrum with the k mesh of $(30 \times 30 \times 3)$ and with band states between (9,116). The laser polarization is linear and along the z axis. To test the convergence with the number of states included, we increase the number of states to (9,144), and we find that the convergence with the number of states is very good, with some minor structure changes as expected, (compare Supplementary Figs. 4(a) and 4(b)).

By contrast, we find that in general, the convergence of HHG signal with the number of k points is more difficult to reach. Supplementary Figure 4(c) shows the results with the k mesh of $40 \times 40 \times 4$, with the same number of band states (9,116). A direct comparison between Supplementary Figs. 4(a) and (c) shows that the harmonics converge very well, with some small differences that would need an additional number of k points. For the current study, we used 9248 processors and spent 24 hours, close to the limit at the National Energy Research Computing Center (NERSC). Therefore, we do not claim that our results are completely converged, but by comparing the maximum momentum expectation values at 0 fs between these two k meshes, we estimate that the error is below 10%. For this reason, we believe that none of our main conclusions is affected by this convergence qualitatively. Interestingly, to the best of our knowledge, none of the prior studies has ever carried out a k point convergence test. Our study fills this gap for HHG investigations.

C. Fe(110) monolayer

1. Laser polarization

We first investigate how the laser polarization affects HHG signals in the Fe(110) monolayer. All other laser parameters are the same, namely, $\hbar\omega = 2$ eV, $E_0 = 0.09$ V/Å, and $\tau = 60$ fs. We use the same k mesh of $30 \times 21 \times 7$. We keep 122 band states, from 17 to 138, which cover all the valence states. The sixteen $3s$ and $3p$ states do not enter our calculation

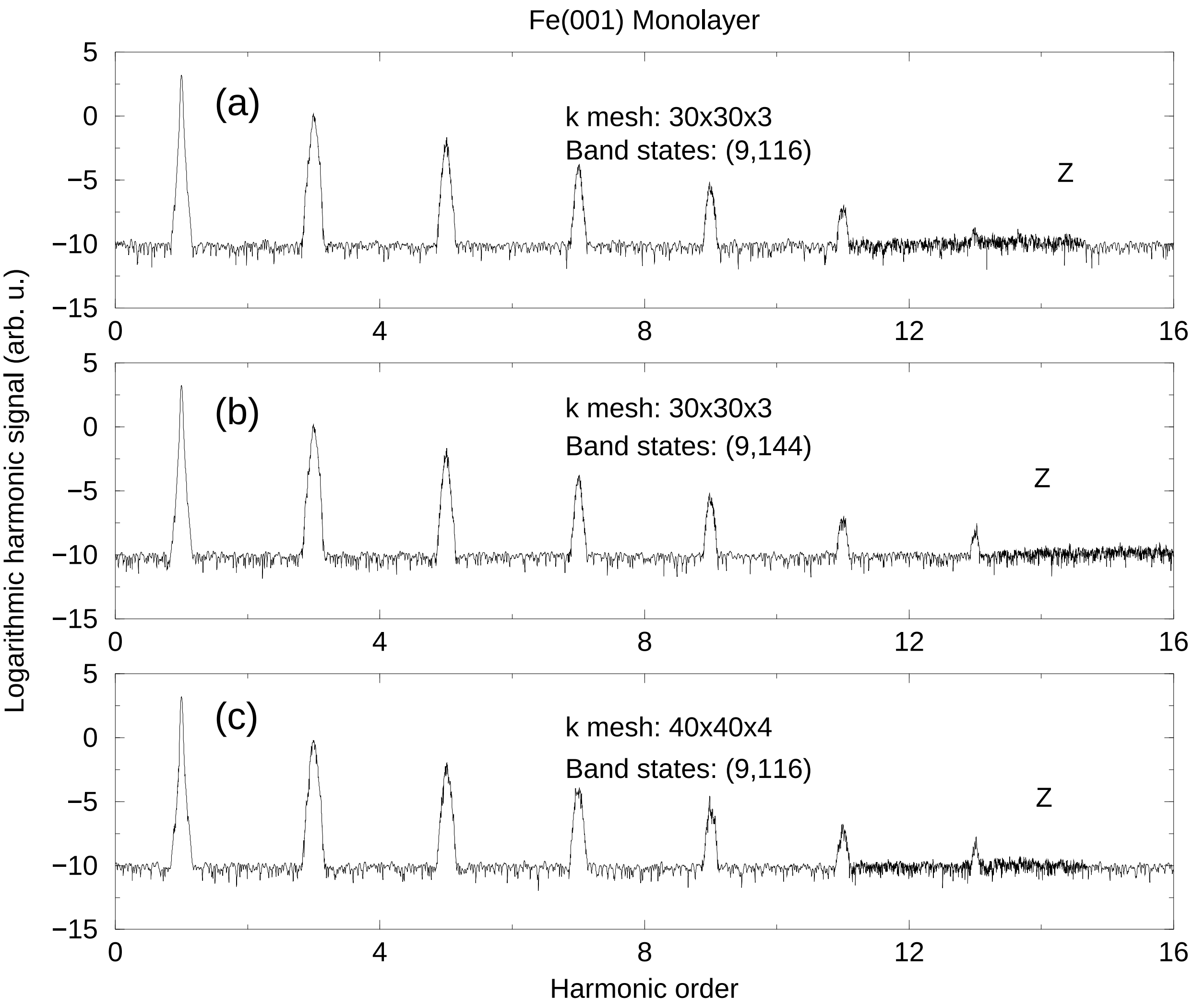

Supplementary Fig. 4: Convergence test for the Fe(001) monolayer. We employ the field amplitude of $E_0 = 0.09$ V/Å, duration of $\tau = 60$ fs, and photon energy $\hbar\omega = 2.0$ eV. The laser polarization is linear and along the z axis. (a) We use a k mesh of $30 \times 30 \times 3$ and include 108 band states, from 9 to 116 at each k point. (b) The same k mesh is used as (a) but with 136 states from 9 to 144. (c) We increase the k mesh to $40 \times 40 \times 4$, while keeping the same number of band states as (a).

for the same reason as above in Fe(001).

Supplementary Figure 5(a) shows the results with the laser polarization linearly along the x axis. The harmonic signals along the x and y axes are denoted by the solid and dotted lines, respectively. We notice that the highest harmonic order is 15th order. The baseline for the x component is higher in general. The situation is reversed when we align the laser

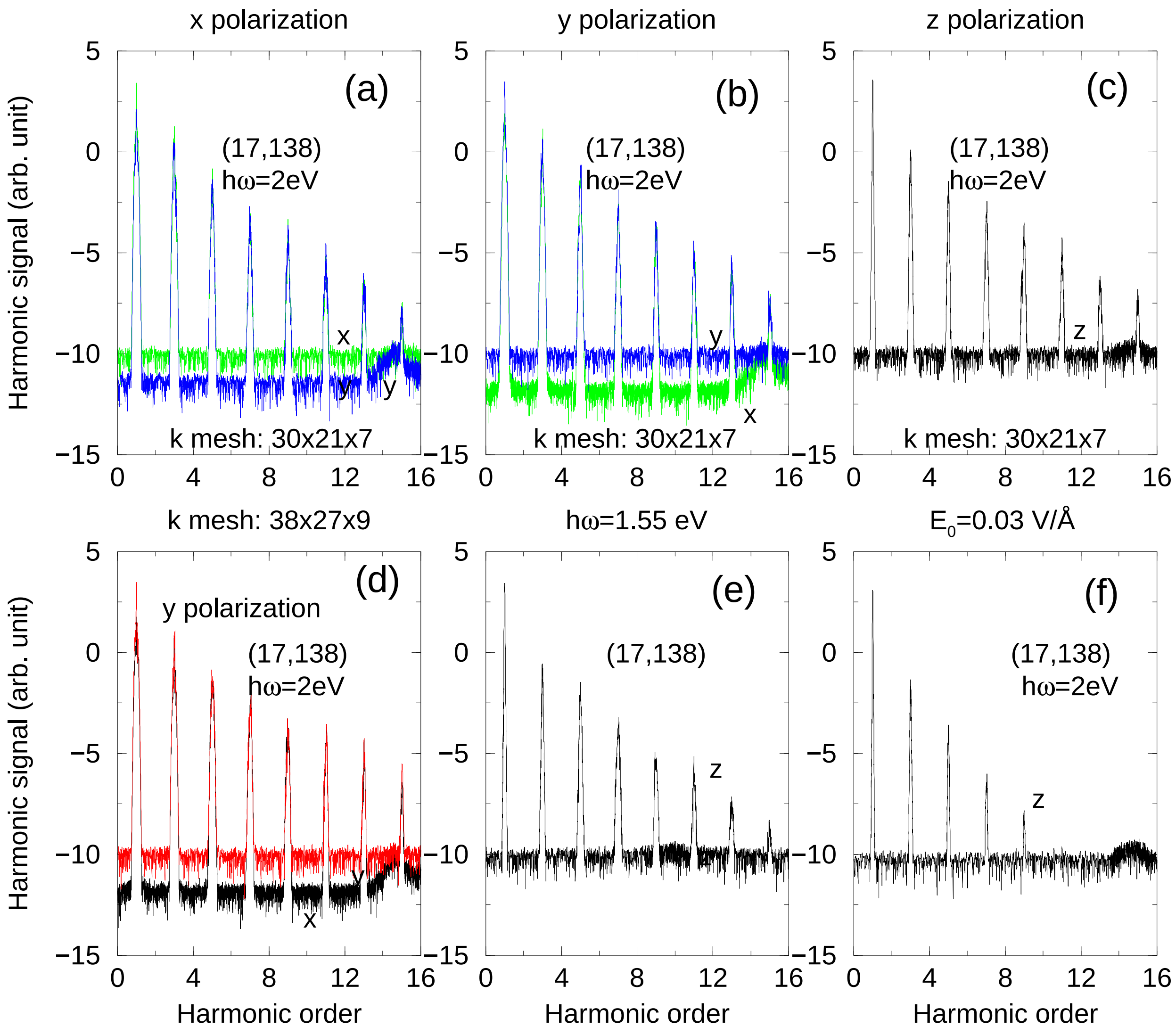

Supplementary Fig. 5: Dependence of HHG in the Fe(110) monolayer on the laser and system parameters. (a) HHG signal with laser polarization along the x axis. The black curve is for the x component, while the red one is for the y component. We include all the states from 17 to 138. $E_0 = 0.09$ V/Å, $\tau = 60$ fs and $\hbar\omega = 2$ eV. (b) Same as (a), except that the laser polarization is along the y axis. Note that the red curve (y component) is higher than the black curve (x component). (c) Same as (a), but the laser polarization is along the z axis. The HHG spectrum is from the z axis. (d) We use a larger k mesh ($38 \times 27 \times 9$). Other parameters are the same as (b). (e) Photon energy is changed to $\hbar\omega = 1.55$ eV. The harmonics are strongly affected. (f) We use a weak field amplitude of 0.03 V/Å.

polarization along the y axis (see Supplementary Fig. 5(b)). For the laser polarization along the z axis, we find that harmonic order also reaches almost 15 (see Supplementary Fig. 5(c)).

2. *k mesh*

Next, we keep the same laser parameters as Supplementary Fig. 5(b), but increase the number of the k points to $38 \times 27 \times 9$. Supplementary Figure 5(d) shows that the overall change in the x and y components is very small, in comparison with Supplementary Fig. 5(b), though the 15th harmonic becomes stronger. We expect that to get a completely converged result with k mesh, a much larger mesh is necessary. Our computing power is already far beyond that employed in the initial investigation of HHG solids by others in in Supplementary reference [2], but is still not enough for the present purpose.

3. *Laser photon energy*

We investigate how the laser photon energy affects the harmonic generation. Supplementary Figure 5(e) is the harmonic spectrum obtained with $\hbar\omega = 1.55$ eV, while other parameters are kept the same as Supplementary Fig. 5(c). Here we see that the harmonic generation strongly depends on the photon energy. The z component is now weaker (compare the 15th harmonic with that in Supplementary Fig. 5(c)). As explained in the main paper, the photon energy may allow one to effectively filter out some band states.

4. *Laser field amplitude*

To demonstrate that the harmonic signal is really from the laser excitation, we use the same parameters as Supplementary Fig. 5(c), but reduce the field amplitude to $E_0 = 0.03$ V/Å. Supplementary Figure 5(f) shows that all the signals are reduced and the highest order is only 7. By contrast, in Supplementary Fig. 2(e) of the paper, when we increase the amplitude to $E_0 = 0.15$ V/Å, we find that the harmonic order reaches 19. This proves that indeed the harmonic generation is due to the laser field.

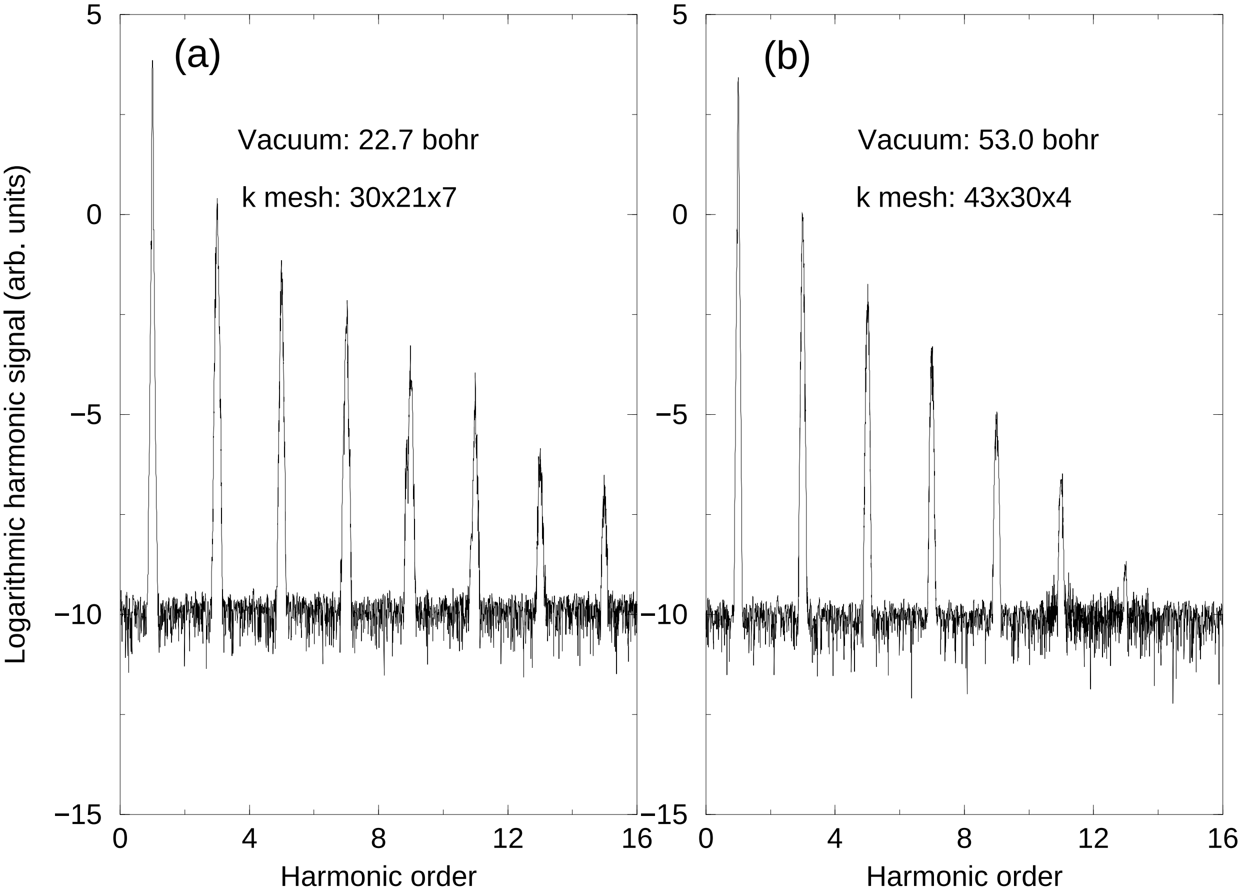

Supplementary Fig. 6: Dependence of HHG signal on the vacuum layer thickness in the Fe(110) monolayer. (a) HHG signal for the vacuum thickness of 22.7 bohr. This thickness is used for our main paper. The k mesh is $30 \times 21 \times 7$. The laser polarization is linear along the z axis. The laser photon energy $\hbar\omega = 2$eV, the field amplitude $E_0 = 0.09$ V/Å and duration $\tau = 60$ fs. (b) HHG signal for the vacuum thickness of 53.0 bohr. The k mesh is $43 \times 30 \times 4$. All the laser parameters are the same as (a).

5. *Vacuum layer thickness*

In the following, we want to test the convergence with the vacuum layer thickness. This is an important test since it affects the results of our calculations, but is a very challenging task since multiple factors, such as the size of basis functions used for different vacuum thickness, intrinsic differences in k mesh grids and different energy cutoff used, potentially make such a comparison difficult. With this in mind, we first show the results for the vacuum layer thickness is 22.7 bohr in Supplementary Fig. 6(a). Supplementary Figure

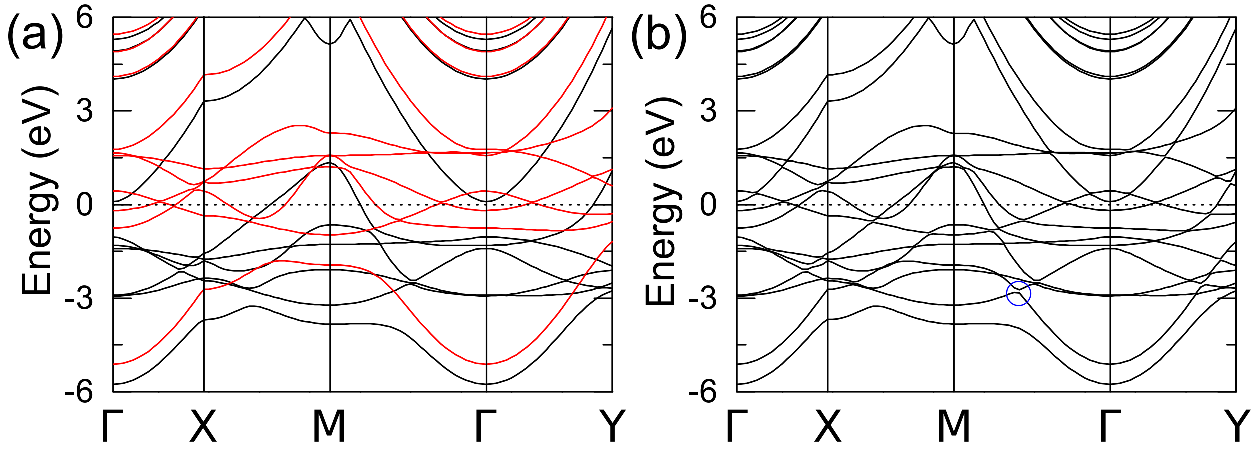

Supplementary Fig. 7: Band structures of the Fe(110) monolayer (a) without and (b) with SOC. The black and red lines in (a) represent the spin majority and minority DOS, respectively. The Fermi level is set to 0 eV.

6(b) shows the results with the vacuum layer thickness of 53.0 bohr, more than twice larger than that in Supplementary Fig. 6(a). We see some differences in high order harmonics, but the difference is smaller for low-order harmonics. This demonstrates that the agreement between these two vacuum thicknesses is satisfactory, given their intrinsic differences and difficulties discussed above. We should also mention that no comparable study on vacuum thickness has been carried out before. Our study represents the first reported effort along this direction.

6. Band structure

In this subsection, we show the band structure of the Fe(110) monolayer. We have performed two calculations, with and without spin-orbit coupling. The results are shown in Supplementary Fig. 7. Supplementary Figure 7(a) shows that the majority band is low on the energy scale (see the black curve), so there are more electrons in these bands. By contrast, the minority bands have fewer electrons, so their contribution to HHG is smaller. Supplementary Figure 7(b) is the band structure with spin-orbit coupling. Here we see that some bands are split due to the spin-orbit coupling. For example, a large band splitting occurs along the M-Γ direction (see the blue circle).

7. *Origin of the 5th harmonic*

Future applications of HHG in solids are directly connected with how much we can interpret and understand those peaks microscopically. They carry the information of our material response to external perturbations. This is particularly important for magnetic materials, as other experimental techniques, such as spin-polarized photoemission, already deliver similar information. As explained above, our calculation includes a band of states from 17 to 128, or 112 band states for each k point. This means that there are 112×112 pairs of transitions that potentially contribute to this single 5th order peak. To eliminate these 12,544 pairs of transitions one by one is very difficult if not impossible. Our first strategy is to eliminate all the one-photon transitions, but this does not work. We find that there is little correlation between the one-photon transition states and the 5th harmonic. After long and hard search, we find a method that can disentangle harmonic peaks efficiently. We work backwards from the harmonic energy. Since we know where the 5th harmonic is situated energetically, we develop a list of those transitions with the transition energy around 10 eV. Since in solids harmonics do not always appear at multiples of the fundamental laser photon energy, we add a small energy window of ± 0.8 eV. Once we shorten the list of potential transitions down to 10 pairs, it is much easier to use the brute force to eliminate each of them pair by pair, while checking whether the 5th harmonic still appears. This finally allows us to pin down the origin of the 5th harmonic. It originates from the radiation from conduction states to one valence state. This valence state is already vacated by laser excitation before this transition is possible. In general, we suggest to separate all the harmonic peaks into two types. Type I, similar to the 5th harmonic above, involves real band transitions. Harmonics in Type I can be used to map band states. Type II involves virtual excitations. Their harmonic peaks appear symmetric with respect to multiples of fundamental photon energy. Harmonics in Type II can not be used to map band states.

8. *Density of states in Fe(110) monolayer*

As explained in our main paper, the density of states plays a key role in HHG. In this section, we compute the partial density of states (DOS) around the Fermi level for the Fe(110) monolayer. Supplementary Figure 8(a) shows the density of states for the spin

up channel. All the curves, except the bottom curve, are shifted up for clarity. The total density of states is plotted on the top. The vertical dashed lines denote the Fermi level which is set at 0 eV. We see that the peak around -2 eV is dominated by the d_{z^2} contribution, while two subpeaks are from the d_{yz} orbitals. There is small density around the Fermi level. Different from the spin up channel, DOS in the spin down channel is across E_f and the main shape is determined by d_{yz} orbital (see Supplementary Fig. 8(b)). Electrons in those occupied states are vital to harmonic generation, since they are mainly responsible for subsequent excitation and radiation. Therefore, the radiation carries rich information about the electronic structure of our system. In the Fe(110) monolayer, the spin up channel plays a dominant role.

D. Comparison between HHG in Fe(110) and Fe(001) monolayers

In the following, we compare harmonic generation for Fe(110) and Fe(001) monolayers on the absolute scale. The k mesh for Fe(110) monolayer is $30 \times 21 \times 7$, while that for Fe(001) monolayer is $40 \times 40 \times 4$. Here the laser parameters are exactly same for both systems, with $\tau = 60$ fs and $E_0 = 0.09$ V/Å. Supplementary Figures 9(a), 9(b) and 9(c) compare harmonic signals between Fe(110) and Fe(001) monolayers with the laser polarization along the x, y and z directions, respectively. The solid curves are for the Fe(110) monolayers and the dotted ones for Fe(001) monolayers. From Supplementary Fig. 9(a), it is clear that the x component in Fe(110) is larger than that in Fe(001). The same trend is also found for the y component (Supplementary Fig. 9(b)). For the z polarization, the ratio in the z component is 2.33 for the first, 2.93 for the third, 3.34 for the 5th, 12.14 for the 7th, 38.01 for the 9th, and 28.05 for the 11th order. There is no signal at the 15th harmonic for Fe(001).

IV. SUPPLEMENTARY NOTE 3: HIGH-HARMONIC GENERATION IN FE(110) AND FE(001) TRILAYERS

Demonstration of high harmonic generation in Fe(110) and Fe(001) monolayers is only a first step toward magnetic high-order harmonic generation. To be sure that what we find here is quite generic, we carry out additional calculations in the Fe(110) and Fe(001) trilayers. These systems are much larger and more complicated. We use the same geometry

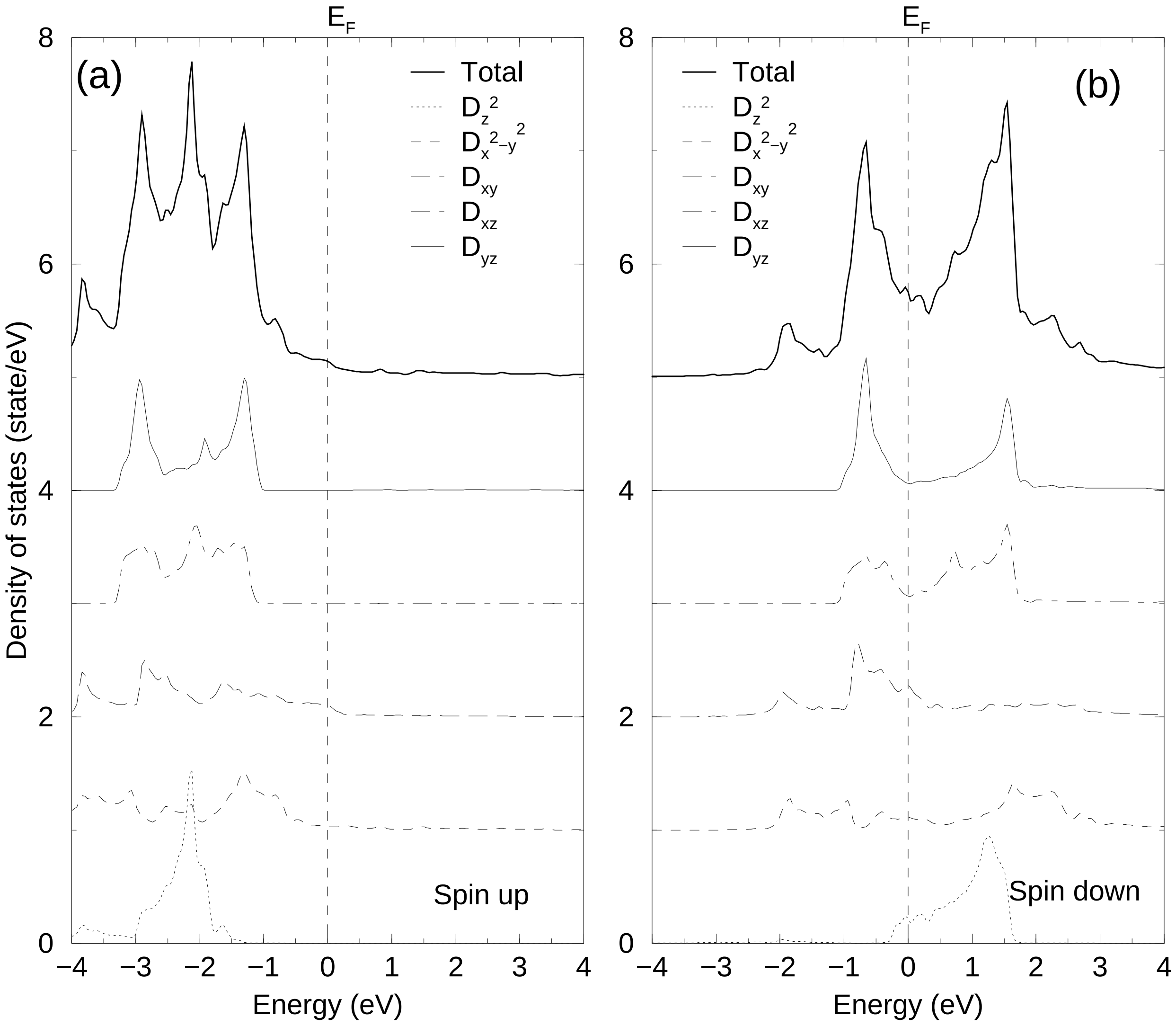

Supplementary Fig. 8: (a) Density of states for spin up channel in the Fe(110) monolayer. The total density of states is plotted on the top (see thick line) and has three major peaks around -2 eV below the Fermi level (vertical dashed line). Except the D_{z^2} partial density of states, all the densities of states are shifted vertically for clarity. (b) Density of states for the spin-down channel. This minority channel has fewer electrons, so their contribution to the HHG signal is smaller.

as that in monolayers. For the Fe(110) trilayer, a vacuum layer of 30 Bohr is added along the slab normal. We use a k mesh of $(35 \times 25 \times 5)$, and the band state cutoff (49, 166) for the same reason as discussed above. We employ the same field amplitude $E_0 = 0.09$ V/Å, the same laser polarization along the z axis and laser pulse duration of 60 fs. Supplementary

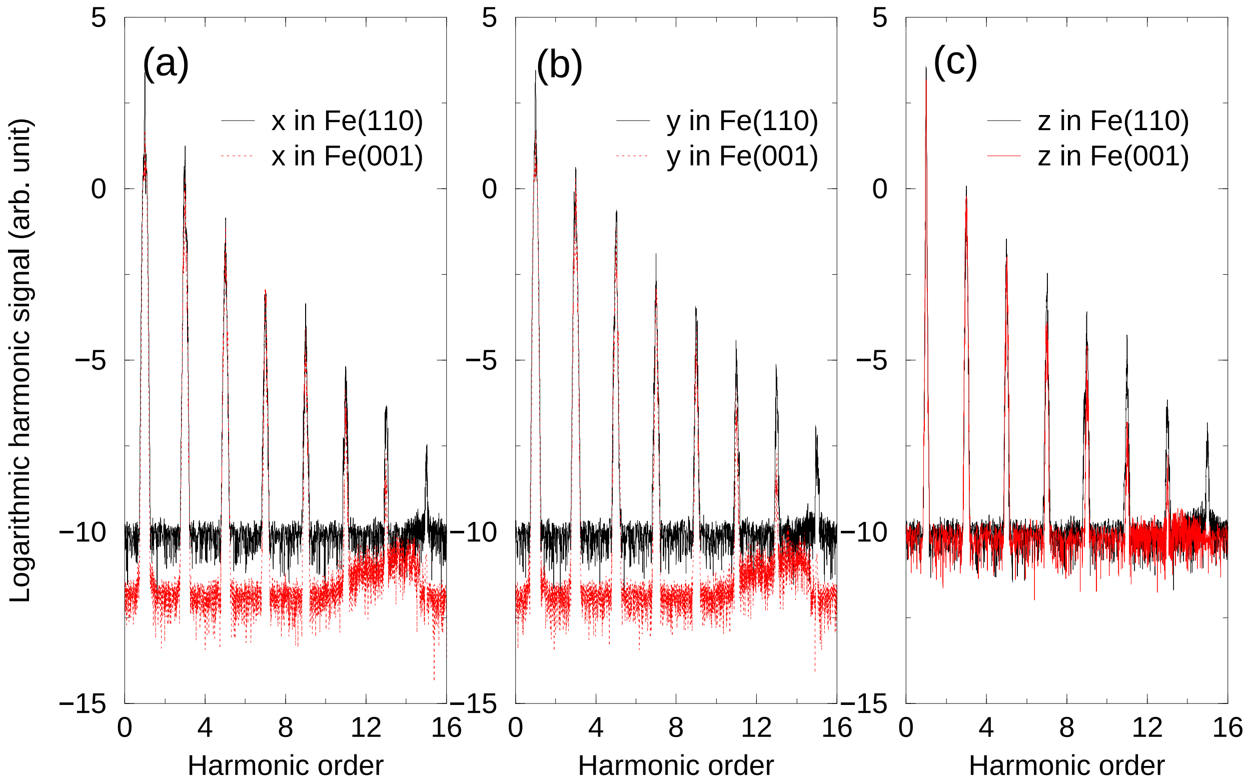

Supplementary Fig. 9: Comparison between HHG signals in the Fe(110) and Fe(001) monolayers with the laser polarization along the (a) x, (b) y and (c) z axes. The solid lines are the results for Fe(110), while the dotted lines for Fe(001). It is clear that harmonic signals are lower in Fe(001) monolayer.

Figure 10(a) shows the harmonic signal for Fe(110) trilayer. We notice that the signal along the z axis is similar to the monolayer case, but the highest harmonic order is 13, two orders lower than the monolayer counterpart. The absolute value of the 13rd harmonic is also lower than that in the monolayer case. We also investigate how the photon energy affects our spectrum. The inset in Supplementary Fig. 10(a) shows the spectra when we use the photon energy of 1.6 eV for two laser pulse durations of 48 fs and 60 fs. It is clear that harmonic generation is not strongly affected by this change.

In the Fe(001) trilayer, we add a vacuum layer of 48 bohr, and use a k mesh of $30 \times 30 \times 3$. The laser parameters are same as those in Supplementary Fig. 10(a). The included band states are from 25 to 122. Supplementary Figure 10(b) is the HHG spectrum for Fe(001) trilayer. One thing that is striking is that the HHG signal along the z axis in the Fe(001) trilayer becomes very similar to that in the Fe(110) trilayer (compare Figs. 10(a) and 10(b)).

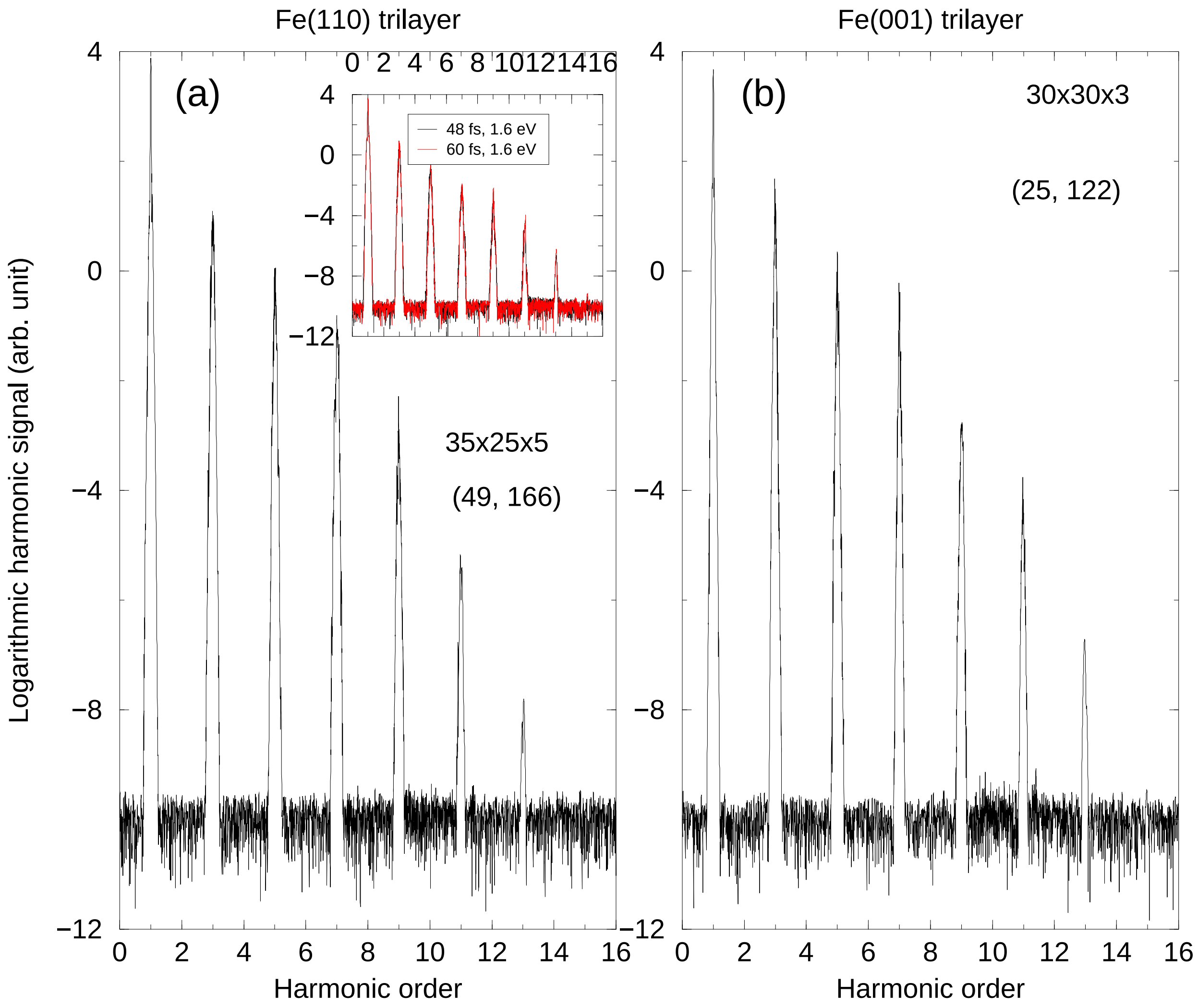

Supplementary Fig. 10: (a) High harmonic generation in the Fe(110) trilayer. The laser polarization is along the z axis. $\tau = 60$ fs, $E_0 = 0.09$ V/Å, $\hbar\omega = 2$ eV. The k mesh is $35 \times 25 \times 5$, and the band states from 49 to 166 are included in our calculation. Inset: HHG signals with the same $\hbar\omega = 1.6$ eV but two different pulse durations of $\tau = 48$ (dotted line) and 60 fs (solid line). (b) High-harmonic generation in the Fe(001) trilayer. The laser parameters are the same as (a). The k mesh is $30 \times 30 \times 3$, and the band states from 25 to 122 are included.

For instance, both have the highest order of 13. In conclusion, together with our results in the main paper, we believe that the high harmonics can be generated from these magnetic monolayers and thin films.

V. SUPPLEMENTARY NOTE 4: COMPARISON BETWEEN FE(110) AND MOS_2 MONOLAYERS

To put the HHG from Fe(110) monolayer in perspective, we also compute the HHG from MoS_2 monolayer (which has two layers). These two systems have different crystal structures. The MoS_2 monolayer has a hexagonal structure and has no inversion symmetry. Its space group is $P\bar{6}m2$ (No. 187) in Supplementary reference [3]. The Mo atom is at (0,0,0), and two S atoms are at (2/3,1/3,z), where z is chosen to be 0.03749706. The lattice constants are $a = b = 5.971537$ bohr and $c = 79.92410900$ bohr. We employ the same laser parameters as those in the Fe(110) monolayer.

Supplementary Figure 11 shows our preliminary results. For clarity, we shift the HHG spectrum for MoS_2 upward by 10. From the figure, we see that the overall harmonic signals are comparable to each other, with harmonics in Fe(110) monolayer slightly higher by 1-2 harmonic orders. MoS_2, due to its lower symmetry and lacking of inversion symmetry, has even-order harmonics.

VI. SUPPLEMENTARY NOTE 5: CRYSTAL-MOMENTUM RESOLVED HIGH-HARMONIC GENERATION

Vampa *et al.* in Supplementary reference [4] proposed high-harmonic generation as a possible band mapping tool. In a semiconductor ZnO crystal, they showed that it is possible to reconstruct momentum-dependent band gaps by exploiting the coherent motion of electron-hole pairs. They argued that this technique is suitable for detection of ultrafast transient modifications to band structures. However, this can not be applied to metallic ferromagnets since the latter have no band gap. We find that for metals it is necessary to categorize harmonics into two types. Harmonics in Type I are associated with virtual transitions and involve nearly free electron gas, where the band structure imprint on the harmonic signal is weak. In Fig. 4(a) of the paper, we show one example of such harmonic. The key feature of Type I harmonics is that their spectra have a symmetric Gaussian shape. These harmonics can not be used to map band structures.

Different from Type I harmonics, those in Type II are associated with real transitions between real electronic band states. They engage real transitions among band states. There-

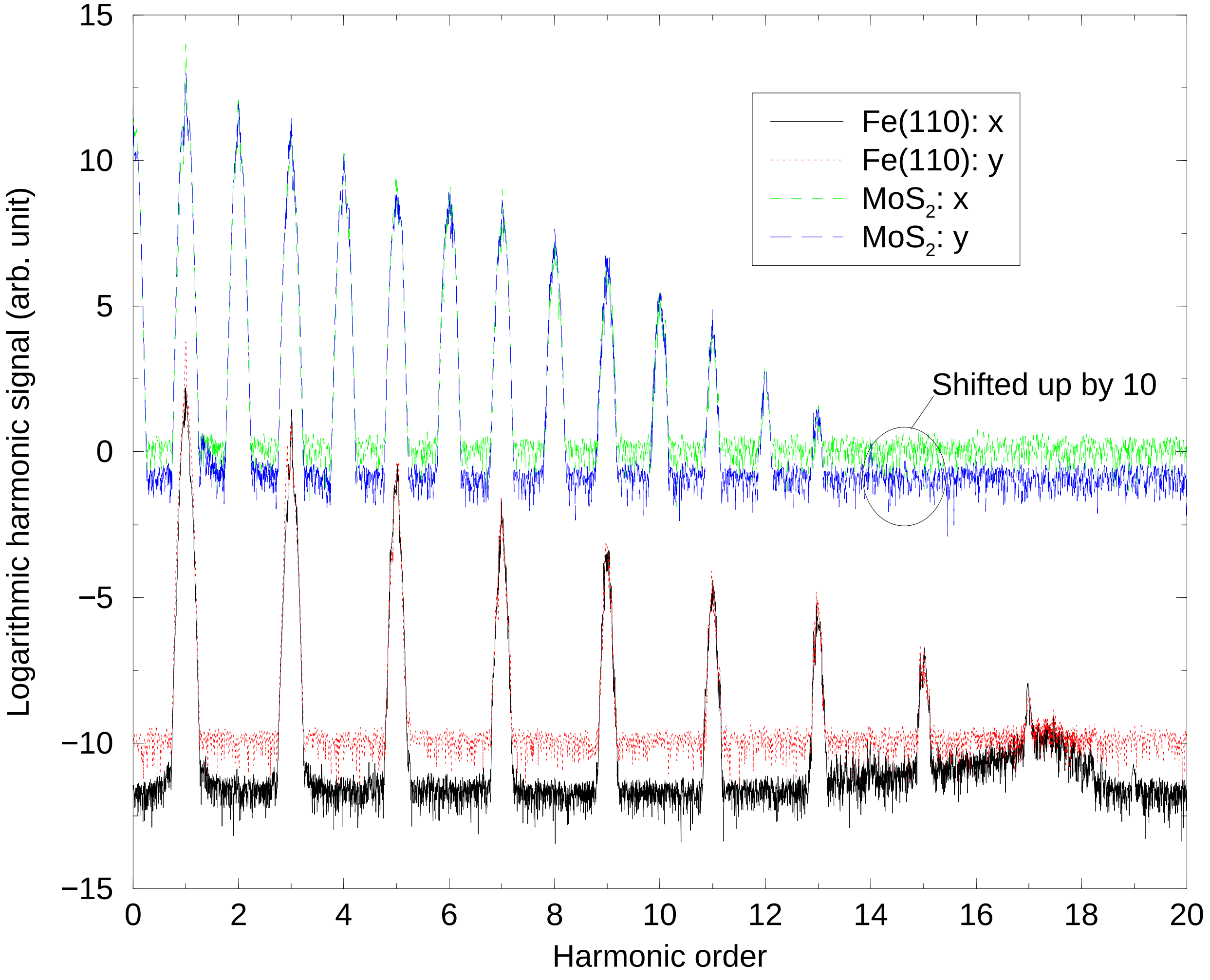

Supplementary Fig. 11: Comparison of the logarithmic high-harmonic spectra for Fe(110) and MoS_2 monolayers. The harmonic spectrum for MoS_2 is vertically shifted by 10 for clarity.

fore, they have the signature of the band structure. For this reason, they can be traced back to the band dispersion. The key feature is that they are normally not symmetric with respect to the harmonic order. In Fig. 4(a) of the paper, we show one example where we disperse the emission along the Γ-Z direction, i.e., Λ line. One sees that those peaks all have an unsymmetric peak with their respective harmonic orders. What makes HHG more powerful is that higher-order harmonics exhibit a stronger dispersion, and different orders of harmonics highlight different parts of band dispersion. This is potentially very useful to future experimental investigations.

VII. SUPPLEMENTARY DISCUSSION: MAGNETIC-DIPOLE EMISSION

Theoretically, even-order harmonics from magnetic dipoles are very interesting by themselves. This combines the beauty of group theory and quantum magnetism theory, and may allow one to probe magnetic properties of a magnet through HHG. It is true that the magnetic-dipole radiation is much weaker. In free ions or atoms, the order of magnitude of the ratio of a magnetic-dipole radiation probability $P(M_1)$ to an electric dipole radiation probability $P(E_1)$ is given by in Supplementary reference [6]

$$\frac{P(M_1)}{P(E_1)} = \left(\frac{\mu}{er}\right) \approx 3 \times 10^{-6}, \tag{6}$$

but we argue that the signal should be detectable for the following reasons. First, for a system with inversion symmetry, the much stronger electric-dipole emission does not contribute to even harmonics. Provided that electric quadrupole radiation is much weaker than magnetic dipoles, the signal from the magnetic-dipole is essentially background free, as odd harmonics are 2 eV (our photon energy) away from the even-order harmonics. The electric quadrupole contribution can be subtracted using two different magnetic field directions, similar to magneto-optics. Second, current experimental detection systems should be able to probe 10^{-6} weaker signals. We take our data in Fig. 2(d) of the paper as an example. Consider the third-harmonic signal as our reference. It has a magnitude of 10^1. Then the second-harmonic signal from magnetic-dipole emission would be at 10^{-5}. Experimentally, Schubert et al. in Supplementary reference [1] used a combination of electro-optic sampling, an InGaAs diode array and a silicon CCD to map out HHG spectra down to 10^{-9}. Similarly, Hohenleutner et al. in Supplementary reference [5] showed in their extended data (their Fig. 1) that their monochromator with a calibrated proelectric detector, a lead sulfide diode, and spectrometers employing InGaAs and cooled Si detectors can probe emission down to 10^{-9}. These two experiments provide strong evidence that those even harmonics from magnetic dipoles should be within reach of current detection schemes. At present, we are looking forward to an actual experiment test. Our results should motivate new experimental activities

in HHG. The potential impact may be beyond our current imagination.

---

[1] O. Schubert, M. Hohenleutner, F. Langer, B. Urbanek, C. Lange, U. Huttner, D. Golde, T. Meier, M. Kira, S. W. Koch and R. Huber, Sub-cycle control of terahertz high-harmonic generation by dynamical Bloch oscillations, Nat. Photon. **8**, 119 (2014).

[2] M. Wu, S. Ghimire, D. A. Reis, K. J. Schafer and M. B. Gaarde, High-harmonic generation from Bloch electrons in solids, Phys. Review A **91**, 04839 (2015).

[3] Z. Y. Zhu, Y. C. Cheng, and U. Schwingenschlögl, Giant spin-orbit-induced spin splitting in two-dimensional transition-metal dichalcogenide semiconductors, Phys. Rev. B **84**, 153402 (2011).

[4] G. Vampa, T. J. Hammond, N. Thire, B. E. Schmidt, F. Legare, C. R. McDonald, T. Brabec, D. D. Klug, and P. B. Corkum, All-optical reconstruction of crystal band structure, Phys. Rev. Lett. **115**, 193603 (2015).

[5] M. Hohenleutner, F. Langer, O. Schubert, M. Knorr, U. Huttner, S. W. Koch, M. Kira, and R. Huber, Real-time observation of interfering crystal electrons in high-harmonic generation, Nature **523**, 572 (2015).

[6] B. Di Dartolo, *Optical interactions in Solids*, (2nd Ed. World Scientific Pub Co Inc; 2 edition, June 30, 2010). Page 369.
